# Effects of gold nanoparticles combined with human β-defensin 3 on the alveolar bone loss of periodontitis in rat

**DOI:** 10.1186/s12938-021-00954-9

**Published:** 2021-11-24

**Authors:** Jing Zhou, Lingjun Li, Di Cui, Xiaoting Xie, Wenrong Yang, Fuhua Yan

**Affiliations:** 1 Stomatology Hospital, School of Stomatology, Zhejiang University School of Medicine, Clinical Research Center for Oral Diseases of Zhejiang Province, Key Laboratory of Oral Biomedical Research of Zhejiang Province, Cancer Center of Zhejiang University, Hangzhou, 310006 Zhejiang China; 2 Nanjing Stomatological Hospital, Medical School of Nanjing University, 30 Zhongyang Road, Nanjing, 210008 Jiangsu China; 3 School of Life and Environmental Science, Centre for Chemistry and Biotechnology, Deakin University, Geelong, VIC 3216 Australia

**Keywords:** Gold nanoparticles, hBD3, Periodontitis, Osteogenesis

## Abstract

**Background:**

Nanomaterials of biomedicine and tissue engineering have been proposed for the treatment of periodontitis in recent years. This study aimed to investigate the effects of gold nanoparticles (AuNPs) combined with human β-defensin 3 (hBD3) on the repair of the alveolar bones of experimental periodontitis in rats.

**Methods:**

A model of experimental periodontitis was established by ligation of the maxillary second molars with silk thread in rats, which were treated with or without AuNPs combined with hBD3. Micro‐computerized tomography (micro-CT) scanning, enzyme-linked immunosorbent assay, and histological and immunohistochemical staining, including alkaline phosphatase (ALP), osteoprotegerin (OPG), tartrate-resistant acid phosphatase (TRAP), and receptor activator of NF-κB ligand (RANKL), were used to analyze the samples.

**Results:**

Micro-CT demonstrated that the alveolar bone resorption was significantly reduced after the treatment with AuNPs combined with hBD3. Levels of TNF-α and IL-6 were decreased markedly compared with the ligation group. H&E and Masson staining showed that AuNPs combined with hBD3 group had less inflammatory cell infiltration, collagen fibrosis and fracture, but higher calcification in the new bone tissue. Moreover, the administration of AuNPs combined with hBD3 increased the expression levels of ALP and OPG (related to bone formation) while decreasing the expression levels of TRAP and RANKL (related to bone resorption) expression.

**Conclusions:**

AuNPs combined with hBD3 had a protective effect on the progression of experimental periodontitis in rats and played a certain role in suppressing osteoclastogenesis and alleviating the inflammatory destruction of periodontitis along with the promotion of bone repair.

## Background

Periodontitis is a chronic infectious disease that occurs in periodontal supporting tissues. As one of the most common oral diseases, it can cause the destruction of the periodontal supporting tissues and eventually contribute to tooth loss [1]. The purpose of periodontitis treatment is to control infection and promote tissue regeneration. However, the effects of tissue regeneration by current periodontal treatments, including scaling and root planing, periodontal flap surgery, and guided tissue regeneration, are limited [2, 3]. In addition, drug therapy is also associated with certain problems, such as drug resistance and flora imbalance, and it is difficult to promote tissue repair and regeneration [4, 5]. Therefore, it is necessary to seek new treatment methods for the disease.

Human β-defensin 3 (hBD3) is one of the most broad-spectrum, antibacterial and cationic defensins in the beta-defensins family [6]. A study in 2018 demonstrated that hBD3 could promote the healing of bacteria-contaminated bone defects in rats [7]. Moreover, Zhu et al. showed that hBD3 might function as an osteogenic promoter to regenerate periodontal tissues in a dog model of periodontitis [8]. However, due to its short half-life and easy hydrolysis characteristics, hBD3 is usually difficult to apply directly. Therefore, the use of new biocompatible materials, such as nanoparticles, has recently been attempted to improve outcomes.

Gold nanoparticles (AuNPs), in view of their ease of synthesis, characterization, and surface functionalization, are recognized as novel nanomaterials in drug delivery, diagnosis, and therapy [9]. Studies have confirmed that AuNPs can be used as anti-inflammatory and antitumor drugs [10, 11]. In addition, AuNPs have always played a pivotal role in bone regeneration engineering. Recent studies demonstrated that AuNPs could promote the repair of alveolar bone defects via cell sheet technology [12]. In addition, AuNPs could regulate the macrophage phenotype to produce a microenvironment with restricted inflammatory cytokine levels and repair cytokines, such as bone morphogenetic protein 2 (BMP-2), thereby promoting periodontal tissue regeneration and preventing the progression of periodontitis [13].

Our previous studies suggested that hBD3 combined with AuNPs could promote the osteogenic differentiation of human periodontal ligament cells [14]. However, the synergistic effects of AuNPs and hBD3 in vivo remain unknown. Therefore, based on the results of previous studies, this study continues to explore whether hBD3 combined with AuNPs can reduce periodontal inflammation and alveolar bone resorption in experimental periodontitis in rats and further investigates the possible mechanism involved in this process.

## Results

### Effects of AuNPs combined with hBD3 on alveolar bone resorption in the SD rats with experimental periodontitis

**Fig. 1:**
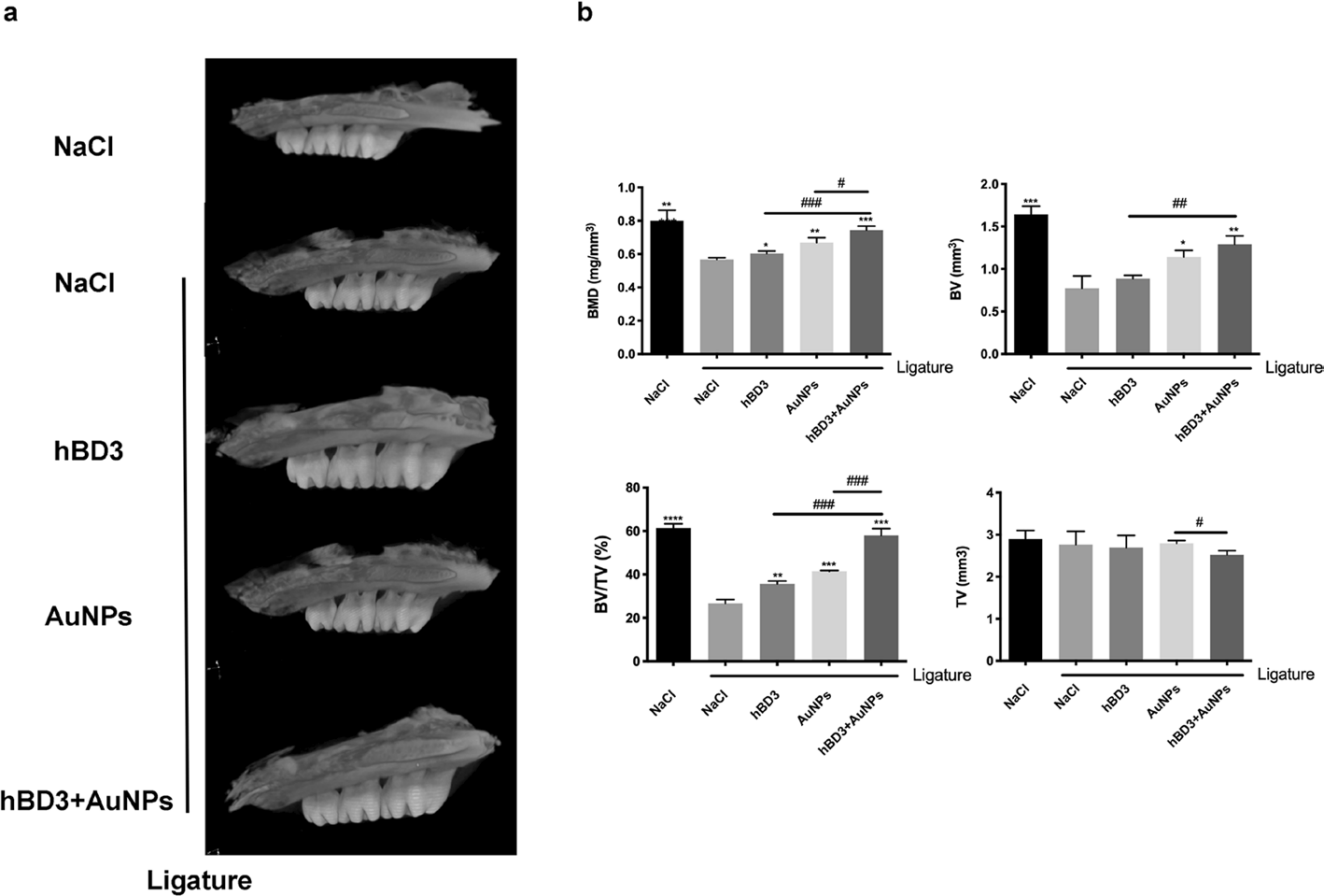
Effects of AuNPs combined with hBD3 on alveolar bone loss. **a** The view of the alveolar bone level was shown by micro-CT through three-dimensional reconstruction images; **b** The BMD (bone mineral density), BV (bone volume), bone fraction (BV/TV) and TV (tissue volume) in the interradicular regions of the second maxillary molars of each group (n = 5 per group). ^*^P < 0.05, ^**^P < 0.01, ^***^P < 0.001, ^****^P < 0.0001, compared with the NaCl + ligation group. ^#^*P* < 0.05, ^##^*P* < 0.01, ^###^*P* < 0.001

The absorption of alveolar bone in SD rats was detected by micro‐computerized tomography (micro-CT). The micro-CT results showed that the alveolar bones of the NaCl + ligation group were significantly absorbed after 14 days, indicating that an experimental periodontal model was successfully established. As shown in Fig. 1a, the degree of alveolar bone resorption of the maxillary second molars in the AuNPs-hBD3 + ligation group was significantly lower than those in the NaCl + ligation group, the hBD3 + ligation group, and the AuNPs + ligation group. In addition, as shown in Fig. 1b, analyses of bone mineral density (BMD), bone volume (BV), and relative bone volume fraction (BV/TV) revealed significantly higher levels in the AuNPs-hBD3 + ligation group than in the other groups, and the tissue volume (TV) of the AuNPs-hBD3 + ligation group was notably lower than that of the other groups.

### Effects of AuNPs combined with hBD3 on serum inflammatory factors in SD rats

**Fig. 2:**
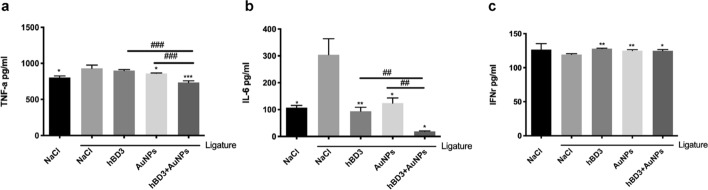
Effects of AuNPs combined with hBD3 on the inflammation profile. ELISA kits were used to measure the serum levels of TNF-α (**a**), IL-6 (**b**), and IFN-γ (**c**). ^*^P < 0.05, ^**^P < 0.01, ^***^P < 0.001, compared with the NaCl + ligation group. ^##^*P* < 0.01, ^###^*P* < 0.001

To further prove the role of hBD3 combined with AuNPs in experimental periodontitis, the concentrations of TNF-α, IL-6, and IFN-γ in serum were detected by enzyme-linked immunosorbent assay (ELISA). As shown in Fig. 2a and b, the expression levels of TNF-α and IL-6 in the NaCl + ligation group were significantly upregulated, indicating that inflammation stimulated the secretion, while the AuNPs-hBD3 + ligation group had significantly reduced serum TNF-α and IL-6 levels. However, as shown in Fig. 2c, the expression of IFN-γ in the NaCl + ligation group was downregulated, while its expression in the hBD3 + AuNPs + ligation group was significantly upregulated.

### Histological examination of rat maxilla treated with AuNPs combined with hBD3

Based on H&E and Masson staining of the rat maxilla, the effects of AuNPs combined with hBD3 on experimental periodontitis were further verified from a histological point of view.

**Fig. 3:**
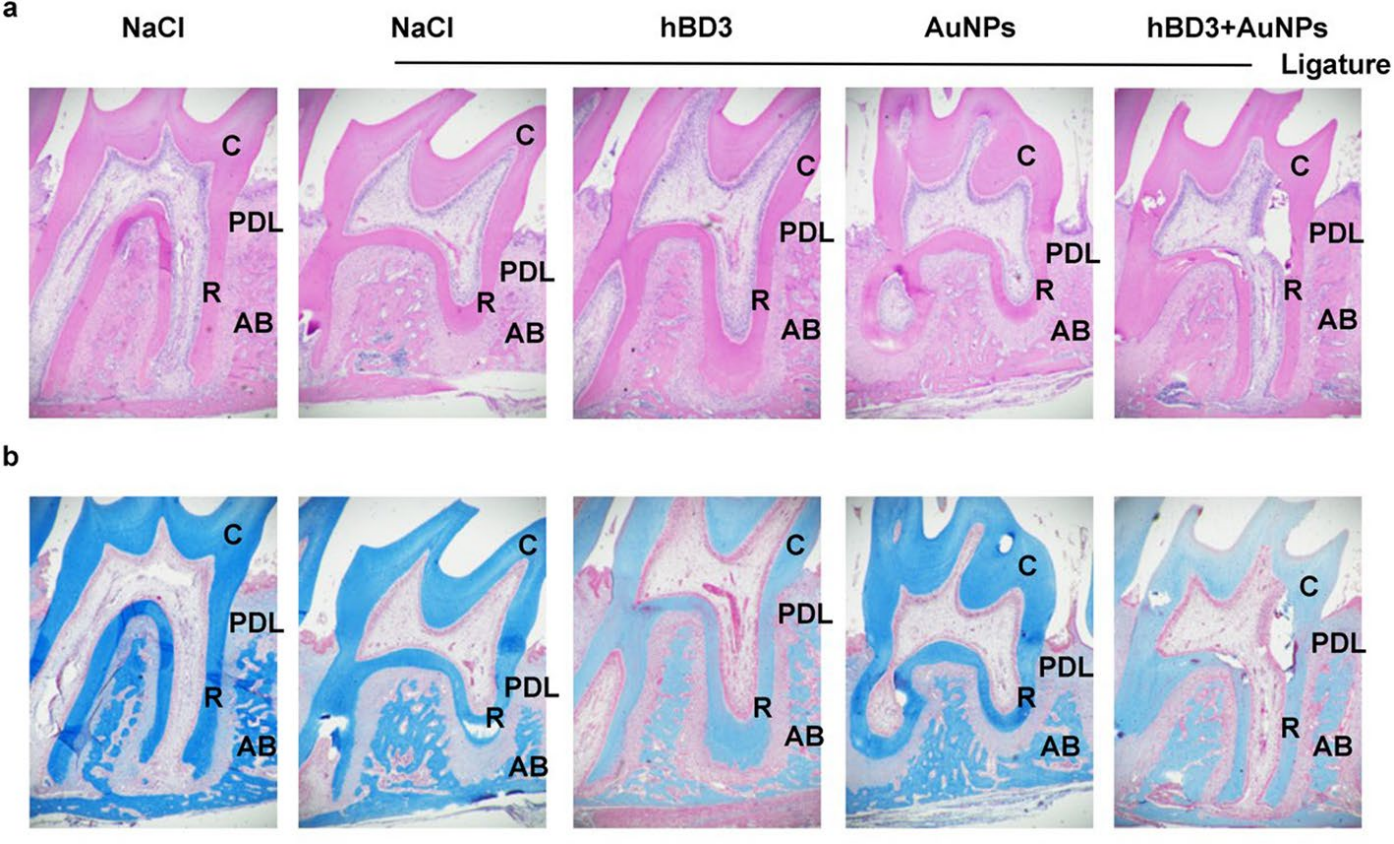
Histological examination of periodontal tissues measured by AuNPs combined with hBD3. **a** H&E staining (× 40), **b** Masson staining (× 40) (C, tooth crown; R, tooth root; AB, alveolar bone; PDL, periodontal ligament)

H&E staining (Fig. 3a) revealed that the periodontal ligament (PDL) in the interproximal area was healthy and that there was no attachment loss in the NaCl group. The NaCl + ligation group showed loss of the interdental papilla and apical migration of the junctional epithelium. Disorganization and disruption of collagen fibers and the presence of moderate to severe mononuclear inflammation in the subepithelial connective tissue and at the margins of the periodontal ligament were also observed. However, the AuNPs-hBD3 + ligation group showed fewer signs of severe inflammation and PDL destruction than the NaCl + ligation group, the AuNPs + ligation group, and the hBD3 + ligation group.

Masson staining (Fig. 3b) revealed that in the NaCl group, the trabecular bone structure was tight, the muscle fibers were stained deeply and obviously, the Havers system structure was obvious, and the bone maturity was high. In the NaCl + ligation group, there were more blue collagen fibers, which indicated that low calcification of the alveolar bone. The Havers system was still in the initial formation stage. However, compared with the NaCl + ligation group, these damages were apparently recovered via combination treatment with AuNPs and hBD3. The Havers system in the new bone tissue was more mature and similar to that of a normal jaw bone tissue structure.

### TRAP and ALP staining of rat maxilla treated with AuNPs combined with hBD3

The effects of hBD3 combined with AuNPs on experimental periodontitis were also determined by observing the numbers of osteoclasts and osteoblasts.

**Fig. 4:**
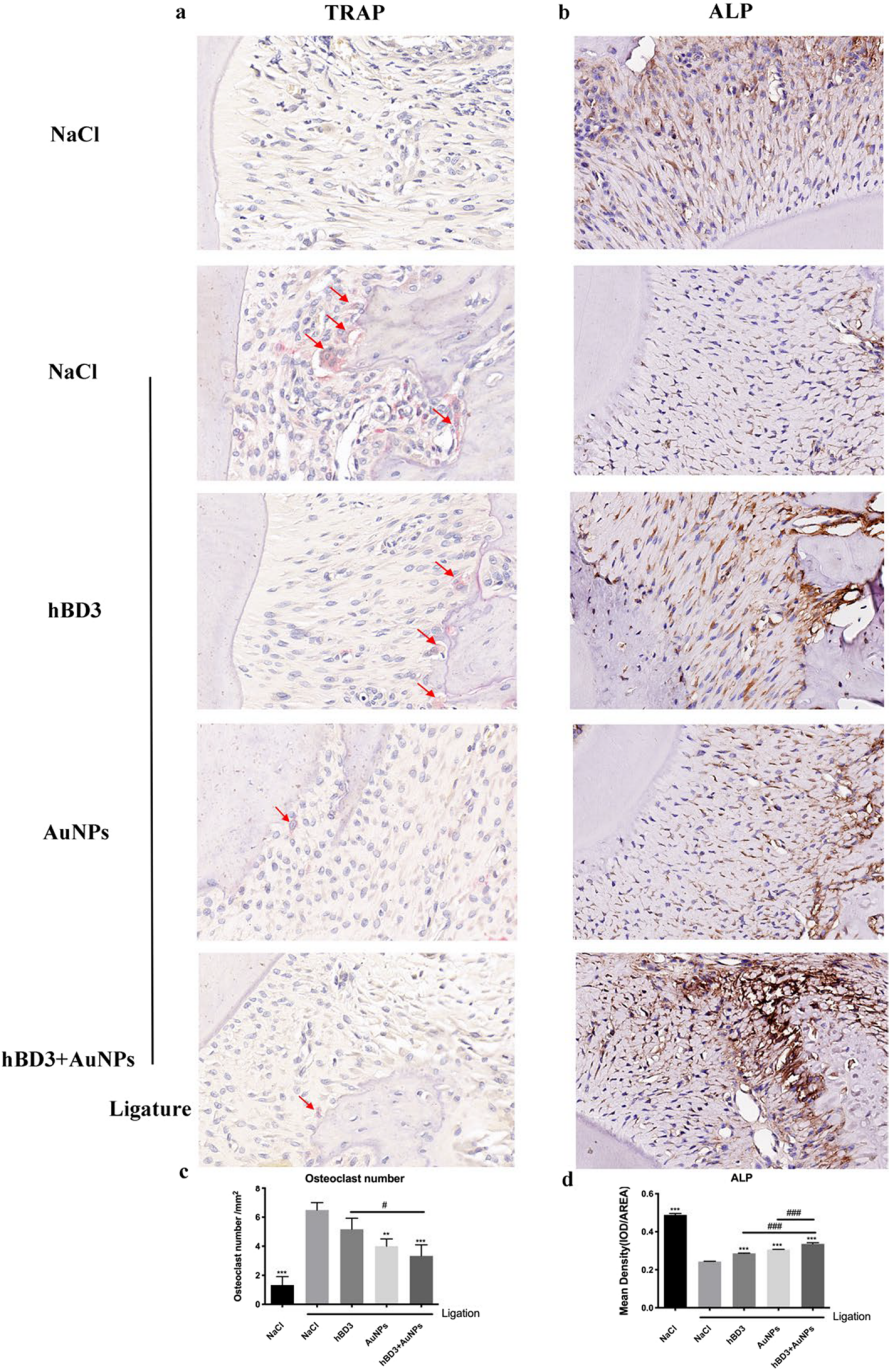
TRAP and ALP staining of periodontal tissues measured by AuNPs combined with hBD3. **a** TRAP staining images (red arrows indicate osteoclasts that were dyed red) (× 400). **b** ALP staining images (× 400). The corresponding quantitative analysis of the osteoclast number (**c**) and ALP expression (**d**) in periodontal tissues. ^***^P < 0.001, compared with the NaCl + ligation group. ^#^*P* < 0.05, ^###^*P* < 0.001

As shown in Fig. 4a and c, there were a number of cells with positive TRAP staining in the alveolar bone in the NaCl + ligation group, indicating active osteoclasts, while there were relatively few TRAP-positive cells in the AuNPs combined with hBD3 group on the surface of the alveolar bone, suggesting that the bone resorption activity was weaker. In contrast, the ALP expression of the AuNPs combined with hBD3 group was markedly increased in the periosteum of the alveolar bone, while it was weak in the control group (Fig. 4b and d).

### OPG and RANKL staining of rat maxilla treated with AuNPs combined with hBD3

Finally, we evaluated OPG and RANKL expression to further confirm the interventional effects of AuNPs combined with hBD3 on experimental periodontitis.

**Fig. 5:**
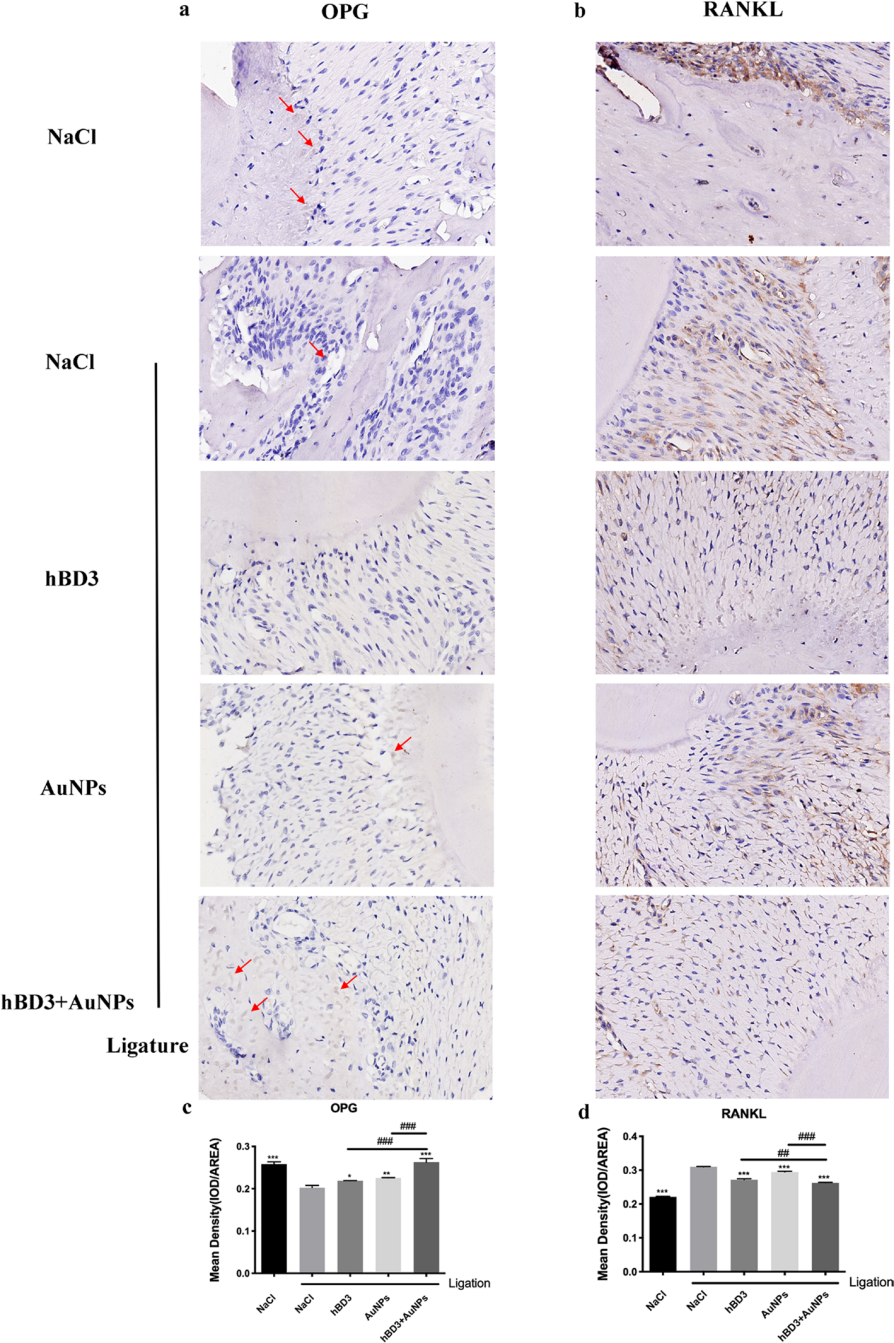
OPG and RANKL staining of periodontal tissues measured by AuNPs combined with hBD3. **a** OPG staining (red arrows indicate osteoblasts that were dyed brown) (× 400) and **b** RANKL staining (× 400). Corresponding quantitative analysis of OPG expression (**c**) and RANKL expression (**d**) in periodontal tissues. ^*^P < 0.05, ^**^P < 0.01, ^***^P < 0.001, compared with the NaCl + ligation group. ^##^*P* < 0.01, ^###^*P* < 0.001

OPG immunostaining showed mild staining in the periodontium of rats in the NaCl + ligation group, while moderate staining (in light brown) was observed, indicating bone formation in the AuNPs-hBD3 + ligation group (Fig. 5a and c).

Compared with the NaCl group, the periodontium of rats subjected to ligation showed marked immunostaining of RANKL (in dark brown). However, AuNPs combined with hBD3 reduced bone resorption in the periodontium of rats subjected to ligation (Fig. 5b and d).

## Discussion

In recent years, several studies have shown that the destruction of periodontal tissue is closely related to the immune response [15]. With the in-depth understanding of the pathogenesis of periodontitis, the interactions among the host immune response, bacteria, and microorganisms and the environment have been found to be crucial in the occurrence and development of periodontitis and the regeneration of periodontal tissues [16]. hBD3 mediated a range of antibacterial biological activities against a broad spectrum of pathogenic microbes by destroying the membrane and forming pores in the plasma membranes of pathogens [17]. In addition, hBD3 can promote periodontal regeneration as sites of infection [7, 18]. However, hBD3 is difficult to widely apply to treatment because of its short half-life and easily hydrolyzed characteristics. AuNPs are widely used as carriers and easily modify the surfaces of polypeptide molecules by physical and chemical techniques, which could compensate for the shortcomings of polypeptide molecules that are easily degraded by proteases and could also take advantage of the carrier to enter cells through receptor-mediated endocytosis [19]. Therefore, AuNPs are considered to be an excellent carrier for the high-efficiency transmembrane transport of extracellular substances. In our study, considering the potential synergistic therapeutic effects of modified AuNPs and peptides, hBD3 was combined with AuNPs and showed more significant effects on the treatment of periodontitis than hBD3 or AuNPs, respectively. Studies have shown that nanoparticles modified with functional peptides can successfully target the cell membrane, enter the cell across the membrane, and even be transported to organelles, such as the nucleus and mitochondria [19–21]. Notably, functional peptides, which play an important role in the interaction between nanoparticles and cells, are also essential to the behavior and distribution of the intracellular transport of nanoparticles [22, 23].

In the periodontal inflammatory microenvironment, lipopolysaccharide (LPS) interacts with monocytes to produce a variety of cytokines that function in classic pathways of periodontitis bone destruction, such as tumor necrosis factor-α (TNF-α) and interleukin-6 (IL-6); it also plays important roles in the progression of periodontitis. In this study, the concentrations of TNF-α and IL-6 in rat serum were determined by ELISA. The results showed that the expression levels of TNF-α and IL-6 in serum increased significantly following ligation. After treatment with AuNPs combined with hBD3, the levels decreased significantly compared with the ligation group, indicating that AuNPs combined with hBD3 could significantly reduce periodontal inflammation. Previous studies confirmed that the expression levels of TNF-α and IL-6 were positively correlated with the severity of periodontitis, which was significantly increased at the site of periodontal inflammation, and its level was significantly reduced after treatment. Therefore, these cytokines could be used as the evaluation index for the degree of periodontal tissue damage [24–26].

The clinicopathological features of chronic periodontitis mainly include the formation of periodontal pockets, alveolar bone resorption, tooth mobility, and tooth loss in the terminal stage. In our study, micro-CT was used to detect the effects of alveolar bone resorption in the rat maxillary second molars. The results showed that there were obvious alveolar bone resorption images at the root furcation of the second molars in the NaCl + ligation group, and alveolar bone resorption was significantly reduced after treatment with AuNPs and hBD3. The analysis of bone parameter-related indicators, including bone mineral density (BMD), bone volume (BV), and relative bone volume fraction (BV/TV), indicated that AuNPs combined with hBD3 effectively contributed to bone formation.

H&E staining showed that compared with the control group, the AuNPs combined with hBD3 group had less inflammatory cell infiltration and less collagen fibrosis and fracture. Masson staining depicted that the AuNPs combined with hBD3 group had obvious red staining and higher calcification in the new bone tissue. Furthermore, ALP and TRAP play a critical roles in osteogenesis as key enzymes involved in the processes of bone matrix deposition and resorption [27]. ALP is unique to osteoblasts and pre-osteoblasts, whereas osteocytes do not secrete ALP, while TRAP is secreted by osteoclasts [28]. In our study, the administration of AuNPs combined with hBD3 increased the expression of ALP while decreasing TRAP expression. Similar effects were observed in MG-63 cells and MC3T3E-1 cells treated with human beta-defensins and gold nanoclusters, respectively [29, 30].

In further research, we continued to explore the mechanism by which AuNPs combined with hBD3 protected periodontitis. During the process of bone reconstruction, osteoclasts and osteoblasts maintain certain numbers to restrict each other and are renewed to keep balance [31]. Osteoprotegerin (OPG) and receptor activator of NF-κB ligand (RANKL) are among the most critical cytokines in this regulation. RANKL is the main regulator of bone resorption. Various cytokines, inflammatory mediators, and hormones indirectly mature and activate osteoclasts mainly by promoting the secretion of RANKL. OPG is the receptor of RANKL; by binding to RANKL, it can hinder the functional activities of RANKL and promote the generation of osteoblasts and the apoptosis of osteoclasts [32, 33]. Therefore, the expression of OPG and RANKL in periodontal tissues plays an important role in regulating alveolar bone resorption [34]. Park et al. showed that HBD3-C15 could inhibit RANKL-induced osteoclast differentiation and disrupt the formation of the RANKL-induced podosome belt [35]. Studies have also shown that AuNPs cannot only suppress pre-osteoclast fusion induced by RANKL and macrophage colony stimulating factor (M-CSF), but also bring about significant downregulation of RANKL gene expression and the RANKL/OPG ratio [36, 37]. Our results demonstrated that AuNPs combined with hBD3 could inhibit the secretion of RANKL and increase the expression of OPG, which might reduce the absorption of alveolar bone.

There are still some limitations in the present study. Osteogenesis is regulated by a variety of cells, factors, and signaling pathways. In this study, we mainly focused on the changes in osteoclasts and the expression of RANKL and OPG in local tissue. However, there has been no in-depth study on the specific cells in the tissue that play roles in alleviating bone resorption and promoting osteogenesis after treatment with AuNPs and hBD3. The specific regulatory mechanisms of the osteogenic differentiation of different cells still need to be further explored.

## Conclusions

Based on the development of nanomaterials and tissue engineering technology, the combination of AuNPs with hBD3 had a protective effect on the progression of experimental periodontitis in rats. We demonstrated that AuNPs combined with hBD3 could suppress osteoclastogenesis and alleviate the inflammatory destruction of periodontitis along with the promotion of bone repair in vivo. These findings suggested that AuNPs combined with hBD3 might be a novel biomedical material in periodontal tissue engineering and periodontitis treatment.

## Methods

### Experimental animals and groups

After being fed adaptively for 1 week, 25 female SD rats at the age of 5 weeks old were randomly divided into five groups (N = 5/groups) as follows: (1) NaCl group, NaCl group with no ligation; (2) NaCl + ligation group, rats with ligature-induced periodontitis alone; (3) hBD3 + ligation group, rats with ligature-induced periodontitis that were treated with hBD3; (4) AuNPs + ligation group, rats with ligature-induced periodontitis that were treated with AuNPs; and (5) AuNPs-hBD3 + ligation group, rats with ligature-induced periodontitis that were treated with AuNPs combined with hBD3. *Reagents and configurations.*

hBD3 was commercially available from Peprotech (Rocky Hill, NJ, USA), and AuNPs with a diameter of 45 nm were synthesized by adopting a chemical reduction method. The concentrations of AuNPs and hBD3 were used according to our published data [13, 14].

### Rat ligature-induced experimental periodontitis model

The rats were anesthetized with pentobarbital sodium (Sigma-Aldrich; Merck Millipore, Darmstadt, Germany) at 50 mg/kg. The silk threads were soaked in medium containing *P. gingivalis* for 2 h ahead of time and were then used to ligate the bilateral maxillary second molars of the SD rats. The control group was treated with no ligation, and the ligated groups were treated with sterile silk thread. According to the groups, 100 μL of 5 μg/mL hBD3, 10 μM AuNPs, or 0.9% NaCl solution was injected into the mesial, central, and distal sides of the maxillary second molars; this process was repeated every 3 days. At the study endpoint, when the rats were 7 weeks old, they were euthanized, blood was collected from retro-orbital, and the maxillary bone, gums, and other tissues were collected and fixed in a 4% neutral paraformaldehyde solution.

### Micro-CT scanning

The maxillary samples of the SD rats were soaked in a 4% paraformaldehyde solution and then were scanned by micro-CT with a Skyscan 1176 scanner (Bruker, Karlsruhe, Germany). The scanning layer thickness was 18 μm, the X-ray exposure time was 404 ms, the tube voltage was 70 kV, and the tube current was 353 μA. After scanning, 3D volume rendering technology from CTVox software was used to convert the 2D CT tomography images into 3D images proportionally and to reconstruct the images to measure the bone mineral density (BMD), bone volume (BV), and tissue volume (TV).

### Detection of serum inflammatory factors

After the rats were anesthetized, the hair around the eyes was cut off, the eyeball was protruding out of the socket, a fine-walled Pasteur pipette (o.d. of 1–2 mm) was inserted into the corner of the eye socket underneath the eyeball, the tip was directed at a 45-degree angle toward the middle of the eye socket. Gentle downward pressure was applied and then released until the vein was broken and blood was visualized entering the pipette. When amounts of blood began filling the pipette, the Eppendorf tube was used to collect blood. After standing at room temperature for 2 h at 2000 × g and centrifuging at 4 °C for 10 min, the serum was transferred to a new centrifuge tube. ELISA kits (R&D Systems, Minneapolis, MN, USA) were used to detect the concentrations of TNF-α, IL-6, and IFN-γ.

### Histological and immunohistochemical analysis

The maxillary samples were soaked in 10% ethylenediaminetetraacetic acid (EDTA) for 4 weeks, dehydrated with an ethanol gradient, and embedded in paraffin. The sections of 5 μm thickness were obtained from sagittal aspects of the second molars. The sections were selected including the mesial and distal roots of the second molar and their root bifurcation. The sections were started to retain from the simultaneous appearance of the roots and root bifurcation of the second molars, until the roots and root bifurcation of the second molars disappeared. Subsequently H&E and Masson staining, as well as the specific tartrate-resistant acid phosphatase (TRAP) staining, was used to evaluate bone resorption and osteoclast activity in the tissue (H&E, Masson, and TRAP staining kits were all obtained from Servicebio, Wuhan, China). The number of osteoclasts per square millimeter around the alveolar bone surface was counted and analyzed on the basis of the TARP staining images. Alkaline phosphatase (ALP) (antibody obtained from Huabio, Hangzhou, China, #ET1601-21, rabbit pAb), osteoprotegerin (OPG) (antibody obtained from Huabio, #EM1701-98, rabbit pAb), and receptor activator of NF-κB ligand (RANKL) (antibody obtained from Servicebio, Wuhan, China, #gb11235, rabbit pAb) expression levels in the periodontal tissues around maxillary second molars were measured by the immunohistochemical method. Stained sections were scanned using CaseViewer software (3Dhistech, Budapest, Hungary). Then, the quantitative analyses of ALP, OPG, and RANKL expression were performed with ImageJ software.

### Statistical analysis

In our study, all statistical computations were performed using GraphPad Prism 6.0 software, and the experimental data of each group are expressed as the mean ± standard deviation (SD). The statistical significance was analyzed using one-way analysis of variance (ANOVA) followed by the Tukey post hoc* test*. When the P value < 0.05, the difference was considered statistically significant.

## Data Availability

All data generated or analyzed during this study are included in this published article.

## Abbreviations

AuNPs: Gold nanoparticles
hBD3: Human β-defensin 3
micro-CT: Micro-computerized tomography
ELISA: Enzyme-linked immunosorbent assay
ALP: Alkaline phosphatase
OPG: Osteoprotegerin
TRAP: Tartrate-resistant acid phosphatase
RANKL: Receptor activator of NF-κB ligand
TNF-α: Tumor necrosis factor-α
IL-6: Interleukin-6
